# A Call for Integrated Approaches in Digital Technology Design for Aging and Disability

**DOI:** 10.1093/geront/gnaf113

**Published:** 2025-03-22

**Authors:** Hanna J Barton, Rupa S Valdez, Ashley Shew, Bonnielin K Swenor, Anna Jolliff, Henry Claypool, Sara J Czaja, Nicole E Werner

**Affiliations:** BerbeeWalsh Department of Emergency Medicine, University of Wisconsin-Madison, Madison, Wisconsin, USA; Department of Systems and Information Engineering, Department of Public Health Sciences, University of Virginia, Charlottesville, Virginia, USA; Department of Science, Technology, and Society (STS), Virginia Tech, Blacksburg, Virginia, USA; Department of Epidemiology at the Johns Hopkins Bloomberg School of Public Health, Johns Hopkins Disability Health Research Center, Johns Hopkins School of Nursing, Wilmer Eye Institute at the Johns Hopkins School of Medicine, Baltimore, Maryland, USA; Department of Anesthesiology, Vanderbilt University School of Medicine, Center for Research and Innovation in Systems Safety, Vanderbilt University Medical Center, Nashville, Tennessee, USA; Lurie Institute for Disability Policy, Heller School for Social Policy and Management, Brandeis University, Waltham, Massachusetts, USA; Division of Geriatrics and Palliative Medicine, Center on Aging and Behavioral Research, Weill Cornell Medicine, New York, New York, USA; Department of Anesthesiology, Vanderbilt University School of Medicine, Center for Research and Innovation in Systems Safety, Vanderbilt University Medical Center, Nashville, Tennessee, USA

**Keywords:** Community engagement, Structural ableism, Structural ageism, Technology design, Technology standards

## Abstract

The fields of aging and disability often proceed as 2 distinct lines of inquiry and action in terms of digital technology design. Guidelines and standards in both spaces (e.g., web content accessibility guidelines) have had suboptimal impact due to limited comprehensiveness enforcement mechanisms. Standards also rarely account for variations within the disability and aging communities and the structural power of ageism and ableism. These concerns proliferate in the context of contemporary technology discourse (e.g., data privacy, generative artificial intelligence). There is an opportunity to bridge both fields given that aging and disability can lead to distinct but overlapping experiences and technological needs and because of the multiple ways aging and disability may be simultaneously experienced. Joint efforts are essential to building the political power necessary to address current limitations and associated harms and to mitigate the risk of exacerbation associated with increasing technological pervasiveness and complexity. Joint efforts can also catalyze a paradigm shift from designing to address “deficits” to designs that are responsive to assets and the context of older adults’ and disabled persons’ full personhood. This paper reviews best practices for digital technology design across aging and disability fields and presents pathways forward toward comprehensive, enforceable standards.

There exist two parallel fields in digital technology design: one focused on aging and the other on disability. These often proceed as distinct lines of inquiry, limiting their potential impact, despite their interrelation. For example, guidelines and standards for designing digital technologies in both spaces have had suboptimal impact due to limited clarity, comprehensiveness, dissemination, and enforcement mechanisms (Arief et al., 2020). Much remains to be done to address digital ageism and digital ableism, and the ways they are interrelated, to fully account for the impacts of structural ageism and structural ableism on technology (Gendron et al., 2024; Mannheim & Köttl, 2024; Shew, 2023). These concerns are likely to proliferate in the context of uncertainties related to data privacy and the increased integration of artificial intelligence into these digital tools.

Digital technology is a prerequisite for full participation across most societal domains. Everything from paying bills and depositing a check to attending a concert or traveling now relies on digital technologies. For the most part, this digital transformation has increased the accessibility of these services. Yet disabled people and older adults experience a growing digital divide (Faverio, 2022; Perrin & Atske, 2021). The failure to effectively design digital technologies for these populations results in their exclusion from relevant everyday activities that contribute to their health and well-being. People who are both older adults and disabled face even more significant exclusion. A future where digital technologies not only include but take innovative approaches to supporting disabled people and older adults hinges on the development of comprehensive and enforceable standards, that is, a set of best practices that are agreed upon by expert consensus. This will ensure the needs of disabled people and older adults are considered throughout digital technology design.

The most meaningful advocacy for the design of digital technologies for these populations will come from building solidarity between the fields of aging and disability. This requires understanding the intersections between aging and disability, aligning models of disability, and cocreating digital technology standards that distill wisdom from both fields. Developing digital technology design standards that can be codified and enforced through policy is critical to ensuring digital technologies afford disabled people and older adults the opportunity to enjoy their human rights.

Standards for the design of digital technologies for these populations will likely need to address both the specific requirements for how digital technologies meet people’s access needs and the processes by which digital technologies are designed, for example, requiring community engagement or user testing. Converging on this first type of standard will be essential for supporting designers who lack the resources to deeply engage community in the design of their digital technologies. The second type of standard, in addition to creating tiered guidelines on how to engage communities in the design and development of digital technologies, may offer much-needed transparency to older adults and disabled people who are looking for technologies that have been specifically designed with them in mind.

By bridging the fields of aging and disability, we can find our way to more comprehensive, enforceable digital technology design standards, creating greater access and better experiences for both disabled people and older adults. In this paper, we review best practices for digital technology design across the fields of aging and disability and present pathways forward, toward comprehensive and enforceable standards.

## Intersections Between Aging and Disability

There is a significant overlap between the aging population and people with disabilities (Bureau, 2024). Among disabled people, 42% are older adults, that is, aging is a critical aspect of many disabled peoples’ experience. And approximately 24% of people aged 65–74 are disabled, increasing to 46% for those over 75, that is, the experience of being or becoming disabled is often characteristic of one’s experience of aging. With the number of older adults (65+) expected to double by 2050 in the United States alone and an increase in disability sparked by global and environmental factors, for example, COVID-19, climate change, and so forth, the number of people represented by the intersection of aging and disability will continue to grow (Bonuck et al., 2024; Gaskin et al., 2017; Hacker, 2024).

Given the growing populations of older adults and disabled people, there is a pressing need to bridge aging-related and disability-related digital technology research and design. This requires creating bridges at multiple levels, starting with models and values, which are then embodied in artifacts such as guidelines and standards.

## Importance of Social Models of Disability and Aging for Digital Technology Design

In designing digital technologies for aging and disability, there is a long history of applying medical models of inquiry, which focus narrowly on biological or physical functioning and pathologize changes in those functions (Brody & Morrison, 2019; Gendron et al., 2024). The medical model is deficit-oriented, focusing on what disabled or aging people cannot do rather than recognizing disability and aging as happening within the social and infrastructural worlds. Disability and aging are natural to human existence and represent experiences that present opportunity for creativity, change, identity, and community. This model limits digital technology development, as it aims to fix perceived medical issues instead of fully including people, regardless of their age or disabilities.

One alternative to the medical model, a social model of disability, acknowledges disability as a normal part of human diversity. In particular, the social model of disability posits that disability occurs when a person interacts with environments and technologies not designed to fit their needs (Lundberg & Chen, 2024), highlighting that disability is not inherent to an individual’s body but involves interactions within a broader context (Guffey & Williamson, 2020; Lawson & Beckett, 2021; Puar, 2015): for example, stairs are problematic, not people unable to use stairs. In a similar vein, nonbiomedical models of aging embrace changes across the life course and point to the importance of engagement, optimism, resilience, and spirituality in facilitating successful aging (Carver & Buchanan, 2016). These models thus address built infrastructure and societal biases that create barriers to inclusion and have been instrumental in civil rights movements, leading to policies like the Americans with Disabilities Act and design approaches like universal design (Guffey, 2018; Hamraie, 2017; Pelka, 2012).

The social model and related models offer valuable insights into the parallels between aging and disability, suggesting ways to align digital technology design within sociotechnical contexts. Unlike the deficit-focused medical model, the social models promote designing technologies that accommodate functional—rather than biological or physical—limitations, fostering innovation and inclusivity. For example, instead of designing digital technologies that center symptom management and medication adherence, we might instead focus on designing digital technologies that account for older adults’ and disabled peoples’ participation in the social sphere through the explicit consideration of how friends, family, or caregivers may contribute to their care, as well as by designing for the variety of environments they may traverse, for example, home, leisure/vacation, community-spaces. Given the social model, we might also begin to consider the social burden(s) the technologies we design may place on people, for example, poorly designed check-in kiosks in Korean and Japanese hospitals making care less accessible for older adults (Park, 2023a). Comprehensive and enforceable digital technology standards grounded in social models of disability and aging can realize this vision.

## Summary of Best Practices for Designing Digital Technologies for Older Adults and Disabled People

The fields of aging and disability technology design are at different places in achieving the goal of having comprehensive and enforceable standards. Currently, research on aging and technology design focuses on understanding digital technology needs of older adults, and individual research groups have started to articulate best practices (Fisk et al., 2020; Gomez-Hernandez et al., 2023). In the disability space, there has been significant movement toward consensus-based standards, for example, web content accessibility guidelines (WCAG), which have been developed and iteratively revised by an international consortium and adopted as policy (with varying enforceability; Caldwell et al., 2008; Cooper et al., 2023).

### Designing for Older Adults

Recommendations for designing digital technologies for older adults often account for differences in sensation, perception, cognition, and other physiological changes such as changes in mobility (Fisk et al., 2020). Research indicates “golden rules” of designing mobile applications for older adults: “simplify the design” and “increase the size and distance” between controls (Gomez-Hernandez et al., 2023). Additional recommendations include simplifying navigation, adjusting visual design, and decreasing the user’s cognitive load. Social locations, such as one’s gender, ethnicity, or race, are also acknowledged as shaping older adults’ relationship to and needs for technology (DeAngelis, 2021; Mitzner et al., 2018). Suggested strategies for understanding these differences include conducting qualitative research to understand the needs of specific subgroups of older adults (Fischer et al., 2020; Mitzner et al., 2018) and conducting usability trials with diverse samples of older adults.

Existing recommendations for designing digital technologies for older adults have garnered some critique (Table 1). First, most recommendations advocating for simplicity do not comprehensively capture the heterogeneity of older adults’ experiences and include ageist beliefs. These recommendations often presume older adults have a generally lower perceptual and cognitive functioning, and disinterest in performing complex tasks using technology, which is not true for all older adults (DeAngelis, 2021). A recent review highlights this presumption, noting studies describing technology design with older adults emphasize “age-related deficiencies” and “technological illiteracy” (Fischer et al., 2020). These recommendations may perpetuate stereotypes of older adults as “frail, incompetent, and vulnerable” by promoting the use of stigmatizing elements (Mannheim & Köttl, 2024; Mannheim et al., 2023).

**Table 1.** Examples of Digital Technology Design Guidance and Related Strengths and Opportunities

Guidance	Strengths	Opportunities for nuance or expansion
Golden rules, for example, “simplify the design” and “increase size and distance” between controls	- Emphasizes the need to design digital technologies for the widest range of perception and cognition	- Some older adults may want to perform more complex tasks with technology. Make digital technologies more customizable
Universal design principles, for example, equitable use, flexibility in use, and simple and intuitive use	- Offers a high-level approach for designing for everyone	- UD doesn’t give clear guidance in many instances, and people often forget to build in flexibility. Develop more detailed guidelines for enacting universal design principles
Web content accessibility guidelines (WCAG)	- Focuses on adjustability to account for a range of needs	- Focus on compliance doesn’t necessarily lead to better usability. Develop greater focus on usability testing, relevant to user experience

Multiple gaps must be addressed to advance toward comprehensive and enforceable digital technology standards for older adults that better acknowledge heterogeneity of experiences, grounded in the social models of disability and aging. These include a lack of recommendations that capture the full range of older adults’ experiences and for the political work of advocating for standards that should be widely adopted as policy. Further, in the United States, current recommendations have been mostly developed in isolation by individual research groups. These groups, along with other constituents, have yet to formally organize and collaborate to propose a set of agreed-upon standards.

### Designing for Disabled People

The most widely known and used digital technology standards for the disability community are universal design principles and the WCAG. Universal design offers principles aimed at making technology more inclusive and accessible. These principles include equitable use, flexibility in use, simple and intuitive use, perceptible information, tolerance for error, low physical effort, and size and space for approach and use (Story, 1998). These principles facilitate accessible technology design, but they are prescriptive only at a high level, and it is often unclear how to implement them. The WCAG international standard begins to articulate how universal design can be operationalized in digital technologies (Caldwell et al., 2008; Cooper et al., 2023). WCAG focuses on guidelines to adapt digital technology to people’s needs with adjustable levers to account for a range of needs rather than assuming one size fits all. For example, rather than recommending making text large, the WCAG indicates text should be resizable.

However, there are limitations to current standards for designing digital technology for disabled people (Table 1). Legal requirements and specifications for design tend to be regarded as an upper limit, rather than a legal minimum, putting the focus on operationalizing in ways that fit with the letter of the law rather than the spirit of inclusion (Cheeseman, 2024). Moreover, current standards focus on certain types of disability such as vision-related disabilities, with less focus on other disabilities such as neurodivergence, cognitive disabilities, and dynamic disabilities (Gartland et al., 2022). Furthermore, standards may not offer plans for implementation, stymying access (Lewthwaite & Swan, 2013).

Additionally, current standards tend to be prescriptive and focused on usability rather than broader experiences of digital technology use, for example, usefulness or relevance of the digital technology to the disabled person. That is, a digital technology can have good usability but still lead to frustration and even harm when it is not designed to fit an individual’s context of use. There is an opportunity to develop additional design standards that foster useful and joyful technology experiences.

A related limitation is the failure of current guidelines and standards to explicitly require community engagement for initial technology design or redesign (Lewthwaite, 2014). The need to engage in a continuous, iterative codesign process applies for technologies fully compliant with standards and those originally using codesign (Papoutsi et al., 2021). Current guidelines and standards are framed in terms of how to design *for* older adults and disabled people (Lewthwaite, 2014). It is essential to emphasize that in addition to creating better standards, it is necessary to move from a paradigm of designing for to designing *with* (Papoutsi et al., 2021). Movements toward “design with disability” and “design with older adults” include greater recognition of disabled people and older adults as critical knowers with expertise necessary to project success, not only test pilots or research subjects after technology has been designed.

## Pathways to Comprehensive and Enforceable Digital Technology Standards

To achieve comprehensive and enforceable digital technology standards, we must close the gap between how the aging-related and disability-related technology research fields view disability. There is a need to move from a hyperfocus on making older adults and disabled people more resilient at a biological level to addressing societal barriers that lead to poor health and social outcomes for these communities. Recognizing the shared experiences between older adults and disabled people may offer a starting point for bridging this gap, especially in technology design.

**Figure 1.**
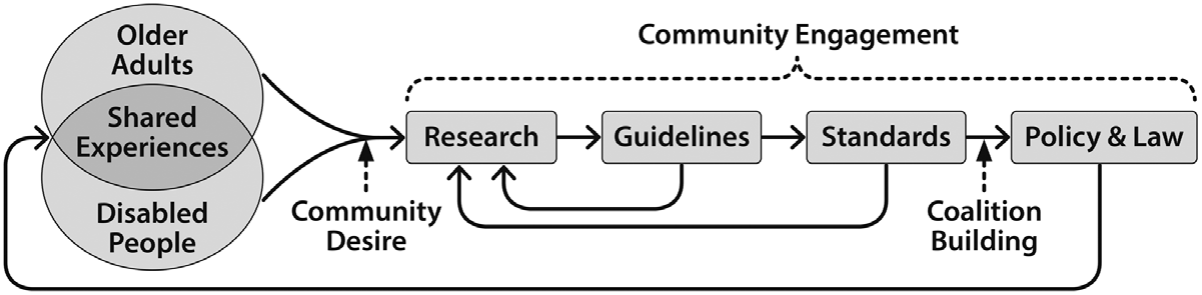
Model for integrating the fields of aging and disability to develop comprehensive and enforceable digital technology design standards.

Our collective goal should be designing a digital technological ecosystem so all can thrive in our current society. To do this, community-engaged research should inform the development of digital technology guidelines, from which standards can be aligned on by groups of experts that include community members, which should then feed into the development of policy and law that codify the inclusion of both populations (Figure 1). There is an opportunity to bridge technology design in aging and disability to facilitate digital technology standards and policy that span both and increase political power to ensure widespread adoption.

### Articulating the Intersection of Older Adults’ and Disabled Peoples’ Experiences

To move toward comprehensive and enforceable digital technology standards that bridge aging and disability, it is critical to first articulate the range of ways that aging and disability intersect. This ensures intersectional experiences are represented within the guideline development process, including:

- Someone born with disability and is aging.
- Someone who has had a disability that uses assistive technology to interact with the world and has had to adapt to new technologies across the lifespan.
- Someone who has had one disability and acquires additional disabilities as they age.
- Someone who has been able-bodied and acquires a disability as an older adult.
- Someone who has had a disability that uses assistive technology to interact with the world and has had to adapt to new technologies across the lifespan.

It is also necessary to account for heterogeneity in older adults (e.g., oldest old, young old) and people with disabilities (e.g., physical, cognitive, sensory, and mental health-related disabilities). It is equally critical to recognize the complexity of individual experience including intersections with other demographic characteristics and identities, especially those associated with health disparities. For people with multiple marginalized identities, for example, a disabled, transgender older adult, ableism, and ageism are necessarily experienced in relation to their other marginalized identities, in this case, their gender identity. Attending to these identities and demographic characteristics and their intersections with aging and disability advances a more holistic understanding of a person’s full experience and implications for technology standards and policies (Valdez et al., 2022).

Achieving this heterogeneity requires measures of disability that capture older adults with disabilities. Current disability measures are limited, as highlighted by recent backlash on proposed changes to how disability is measured by the United States Census Bureau (Landes, 2023). Current methods of measuring and estimating disability are not robust and do not identify people across the full range of disability across the lifespan.

Even when disability data are collected, some of the most widely adopted tools do not identify all disabled people (Bureau, 2024; Statistics, 2024). For example, the American Community Survey does not include learning disabilities, upper extremity disabilities, or dynamic disabilities. Further, civil rights laws such as the Americans with Disability Act (ADA) outline that someone can have a disability and not be experiencing functional limitations; for example, someone with bipolar disorder or Crohn’s disease whose symptoms are controlled with medication. These questions also do not account for acquired disabilities across the lifespan, which meaningfully shapes someone’s experience with disability and their experience with technology. Moreover, many people who have functional limitations do not self-identify as disabled, in part because of pervasive and persistent stigma. To address disability data limitations, we must develop improved methods to collect disability data, ensuring that older adults with disabilities and people who count as disabled even if they don’t identify that way are included. Failure to identify the range of people who are older adults and disabled leads to erasure of these experiences and restricts the ability to create technology standards that are responsive to all needs.

### Engaging Communities in Research and Digital Technology Design

Once we have more comprehensive data to identify people across experiences of aging and disability, we need to create a wide range of mechanisms to gather feedback and understand different experiences of digital technologies to synthesize into technology guidelines and standards. Some of this work might be done through advisory boards, coinvestigation, and research participation (Barton et al., 2025; Harris et al., 2024). Such mechanisms for feedback should allow for input as it relates to digital technology design, technology implementation and maintenance, and a wide range of outcomes experienced during its use (Valdez et al., 2021; Veinot et al., 2018). The disability justice movement principle “leadership of the most impacted” suggests that beyond community engagement, actively empowering and amplifying the voices of the most marginalized members of our communities is essential to ensuring our digital technologies do not further marginalize people with intersectional identities (Piepzna-Samarasinha, 2018).

Directly including disabled people, older adults, and disabled older adults in the design process is a critical strategy for creating technologies tailored to and usable for different social locations (O’Brien et al., 2021; Sumner et al., 2021). Currently, most studies aiming to include older adults or disabled people in technology design do so as research participants, and researchers retain significant power over final design decisions (Valdez et al., 2022; Sumner et al., 2021). Approaches that conceptualize end users’ contributions as the driving force behind design decisions, rather than as selective participation, are more likely to result in more useful, more beneficial, and more accessible designs (Tong et al., 2022).

Engagement should synthesize the tenets of community-based participatory research and codesign (Israel et al., 2005; Unertl et al., 2016). Such efforts should proceed in ways that are nonableist and nonageist (Mannheim et al., 2023; Valdez et al., 2022), and are fully representative of the heterogeneity of both communities. For example, using maxim variance sampling within a specific study or to focus on a specific subcommunity in depth but being attentive to intersectionalities within that community (Benda et al., 2020). Further, at a national level, working with funding agencies to ensure maximum representation within a portfolio of funded research. The engagement practices used need to be intentionally inclusive across the design process including bringing disabled older adults onto the design team and ensuring that those who are members of the community have equal, if not more power in the process.

We can build on current efforts within individual research groups to understand these needs across the design life cycle by creating mechanisms for collating and synthesizing this work. Synthesis of such knowledge ensures that knowledge does not remain hard to access and is presented in a way that is easily understandable. Importantly, synthesizing this knowledge allows for our digital technology design guidelines and standards to be more effectively based upon empirical evidence. This approach would also allow us to clearly identify what gaps remain and what additional engagement is needed (Mannheim et al., 2023).

### Developing Standards and Enforceable Policy

The digital technology design standards developed to integrate the diverse needs of older adult and disabled communities can thus offer a framework for ensuring their inclusion in society, but only if those standards are effectively codified and enforced through policy and law. It will be essential, as the nature of our digital technologies change to iteratively develop design guidance through the aforementioned community engagement mechanisms. Thus, standards, too, should be iteratively reassessed and revised to remain relevant in the current digital technology landscape.

We acknowledge that it is not straight forward to develop standards that are comprehensive, yet specific enough to address the needs of all people. However, we believe the answer lies in developing standards of different kinds and with sufficient iteration and feedback mechanisms. For example, standards may include both recommendations for specific functionalities of digital technologies, and also recommendations for effectively engaging community in the design process. We look to the ways in which the Food and Drug Administration (FDA) has implemented usability testing requirements for medical devices to minimize potential use errors and resulting harm as a model for how to develop standards (and translate those standards into policy) in ways that prioritize safety without hindering innovation (FDA, 2016).

### Coalition Building for Awareness and Political Power

Coalition building is required to have the most robust engagement processes; to create comprehensive and enforceable standards; and to effectively advocate for policies needed to ensure standards are widely adopted and enforced. Coalition building is necessary within and across aging and disability spaces including research communities, community organizations, advocacy organizations, and technology developers.

Coalitions can build on current momentum to raise the overall awareness that the problem is not the person using digital technology but rather the problem is limitations in the way technology is designed, implemented, and evaluated. As it is not uncommon for both older adults and disabled people to have internalized ageism or internalized ableism and thus to also believe that the problem is individual rather than structural (Gendron et al., 2024), awareness is key not only for those who currently have power to create better standards and associated policies but also for members of the community who might not see themselves as oppressed groups.

Disability is a normal, natural part of human life and inherent to the human condition (Garland-Thomson, 2012), just as much as aging is, and becoming disabled can be a common aspect of aging. Young people can be disabled and older people can be nondisabled, too. When it comes to how we address each in society, older adults and disabled people often engage the same mechanisms: healthcare, therapeutic intervention, social organizing and lobbying, community care, social infrastructure, infrastructure (like ramps), and technology development, but we do so in a bifurcated way. Older adults may not see themselves as represented in the disability community because of ableist beliefs, misunderstanding, and lack of representation (Van der Horst & Vickerstaff, 2022), and disabled people can also be ageist. A perpetuated segregation of our communities hinders collective action (Gendron et al., 2024).

Because of these cultural values, structures have been created that reinforce the separation between aging and disability. For example, government organizations separately provide services to both communities and separately provide research funding for both communities, and advocacy organizations and research groups arise to separately represent each. The distinct division in U.S. research funding for aging-related and disability-related technology research further enforces an artificial divide between researchers and designers that might otherwise have overlap. Given that policy development relies on evidence generated by these research fields, disparate funding continues to result in policies and protections for older adults that do not necessarily consider the overlap in needs of the disabled community.

It is essential to integrate these organizations and efforts to bridge knowledge gaps, facilitate mutual learning, generate new learning at the intersection of aging and disability, and to build the political power necessary to effect change. This bridging is what is needed to counter other forces such as barriers to policy making that stem from corporate interests and the pervasive stigmas of ageism and ableism.

### Toward Implementation and Evaluation

In both the aging and disability spaces, guidelines and standards are typically focused on digital technology design with little attention paid to implementation and evaluation. Focusing on just this aspect of the design lifecycle is problematic as the technology could be perfectly designed and yet implemented in ways that hinder full accessibility. For example, if a technology allows for synchronous communication through American Sign Language (ASL), it is only usable for someone who relies on ASL if an ASL interpreter is consistently available as part of the technology. Moreover, without customer service that can guide users through all accessibility features, the technology may remain inaccessible (Valdez et al., 2021). It is essential to monitor outcomes and in particular any unintended consequences of implementation such as intervention-generated inequities, that is, emergent impacts of an intervention which have disparate impacts and may produce inequitable outcomes (Veinot et al., 2018).

### Envisioning a Future of Joyful Digital Technologies for Older Adults and Disabled People

Both older adults and people with disabilities have been segregated, and technology design has been a part of this manifestation of structural ageism and ableism (Levy, 2022; Shew, 2023; Valdez & Swenor, 2023). Technology has the power to promote stigma or to dismantle it. Thus, digital technology design standards should promote positive and even joyful experiences with technology. We can reject drudgery and lack of functionality. There are examples of this in technology and devices for younger people—where hearing aid covers, and ostomy bag covers can be colorful. These designs could be seen as rejection of stigma with an “in your face” attention-draw and a rejection of stigmatizing colors that promote invisibility such as hearing aids in taupe, gray, and brown (Mannheim & Köttl, 2024; Park, 2023b). Although there have been some efforts to bring joyful experience by reimagining what technologies designed for disabled people and older adults could look like, such efforts remain limited and often in the realm of imagining futures. For example, although criticized for lack of inclusion of the disability community in its curation (Jackson, 2023), an exhibit at Cooper Hewitt museum showcased possibilities of how both a range of existing and futuristic technologies could promote self-expression and joyful experience with technology in everyday life (Smithsonian, 2017).

## Conclusions

Creation of comprehensive and enforceable digital technology design standards requires bridging the fields of technology design for older adults and for disabled people, which would benefit both communities. There is an opportunity to bridge both fields given that aging and disability can lead to distinct but overlapping experiences and technological needs and because of the multiple ways in which aging and disability may be simultaneously experienced. Such joint efforts can also catalyze the design of technologies that shift the paradigm from designing to address “deficits” to designs that are responsive to spaces of abundance in the context of older adults’ and disabled persons’ full personhood. Such joint efforts are essential to building the political power necessary to address current limitations and associated harms and to mitigate the risk of exacerbation associated with increasing technological pervasiveness and complexity.
